# 

**Published:** 1875-11

**Authors:** 


### Books and Pamphlets Received.

A Text-Book of Human Physiology; designed for the use of Practitioners and Students of Medicine. By Austin Flint, Jr., M. D. New York: D. Appleton & Co., 1875. Buffalo: Martin Taylor.

A Treatise on Therapeutics, comprising Materia Medica and Toxicology; with especial reference to the application of the Physiological action of Drugs to Clinical Medicine. By H. C. Wood, Jr., M. D. Second Edition; Revised and Enlarged. Philadelphia: J. B. Lippincott & Co., 1876. Buffalo: Martin Taylor.

Lectures on Diseases of the Nervous System. By Jerome K. Bauduy, M. D, Philadelphia: J. B. Lippincott & Co., 1876. Buffalo: Martin Taylor.

A Practical Treatise on Fractures and Dislocations. By Frank Hastings Hamilton, A. M., M. D., LL. D. Fifth Edition. Philadelphia: H. C. Lea, 1875. Buffalo: T. Butler & Son.

Phithisis; its Morbid Anatomy, Etiology, Symptomatic Events, and Complications, Fatality and Prognosis, Treatment and Diagnosis. In a series of Clinical Studies. By Austin Flint, M. D. Philadelphia: Henry C. Lea, 1875. Buffalo: T. Butler & Son.

Hints in the Obstetric Procedure. By William B. Atkinson, M. D. Philadelphia: 1875.

Transactions of the Medical Society of the State of Pennsylvania, at its Twenty-Sixth Annual Session, June, 1875.

Twenty-Second Annual Report of the Trustees of the State Lunatic Hospital, Taunton, Mass.

Manitou, Colorado: Its Mineral Waters and Climate. By S. Edwin Solby, M. R, C. S. 1875.

The American Journal of Microscopy and Popular Seience. Vol. I, No. I. New York, December, 1875.

### Contents of November Number.

### ORIGINAL.

THE BUFFALO

### Medical and Surgical Journal

PUBLISHED MONTHLY,

Containing Original Articles, Reports of Medical Societies and Hospitals, Editorial, Reviews, Correspondence, Army News, etc., etc ; including the usual variety of Medical Periodical Publications. Specimen copies sent on application.

Address Buffalo Medical & Surgical Journal, Buffalo, N. Y.

TERMS OF SUBSCRIPTION, - $3.00 PER YEAR, IN ADVANCE.

